# Ankle biomechanics of the three-step layup in a basketball player with chronic ankle instability

**DOI:** 10.1038/s41598-023-45794-w

**Published:** 2023-10-31

**Authors:** Luyu Wang, Jiahui Ye, Xuyang Zhang

**Affiliations:** China Basketball College, Beijing Sport University, Beijing City, 100084 China

**Keywords:** Health care, Occupational health

## Abstract

At present, the effects of chronic ankle instability (CAI) on the biomechanics of the ankle joint in the three-step layup of basketball players are not clear. This work aims to thoroughly investigate the impact of CAI on the biomechanical characteristics of the ankle during the execution of a three-step layup in basketball players. Thirty male basketball players were stratified into distinct groups—namely, a CAI group and a non-CAI group—comprising 15 individuals each, based on the presence or absence of CAI. Demographic attributes, including age, weight, height, and the Cumberland Ankle Instability Tool (CAIT) score, were subjected to rigorous statistical examination within both athlete cohorts. The research employed four Whistler 9281CA 3D force measuring platforms (Switzerland), recording at 1000 Hz, in conjunction with eight camera motion analysis systems (USA), functioning at a frequency of 200 Hz. The study recorded maximal plantarflexion angle, inversion angle, dorsiflexion angle, and peak ankle dorsiflexion moment across the subjects during the distinct phases of push-off, landing, and the ensuing landing period. The findings notably exhibited that within the context of the one-foot push-off phase, the maximum ankle inversion angle was notably diminished in the CAI group as contrasted with the non-CAI group, demonstrating statistical significance (t = − 3.006, *P* < 0.01). The CAI group exhibited a lesser alteration in ankle inversion angle compared to the non-CAI group. Notably, during the one-foot landing period, the CAI group demonstrated a significantly greater maximum ankle inversion angle in contrast to the non-CAI group (t = 8.802, *P* < 0.001). Furthermore, the CAI group displayed a substantially larger maximum dorsiflexion angle at the ankle joint compared to the non-CAI group (t = 2.265, *P* < 0.05). Additionally, the CAI group exhibited a prolonged peak time for ankle dorsiflexion moment as compared to the non-CAI group (t = − 2.428, *P* < 0.05). Collectively, the findings elucidated a reduction in the maximum ankle joint inversion angle during the one-foot push-off phase in individuals with CAI. Furthermore, increased maximum inversion angle and maximum dorsiflexion angle of the ankle joint were observed during the one-foot landing period, alongside a lengthening of the peak time of ankle dorsiflexion moment. These results contribute valuable insights into the selection of training methodologies for basketball players afflicted by CAI.

## Introduction

The rapid evolution of basketball has spurred continuous enhancements in players’ athletic abilities and physical confrontation capacities, concurrently augmenting the likelihood of player injuries^1^. Within the realm of basketball, the frequent actions of jumping and landing impose escalated loads upon the ankle joint^2,3^, which stands as a primary site of injury^4^. Research findings reveal that 78% of basketball-related injuries occur in the lower extremities, with ankle injuries accounting for 48% of these incidents^5^. Pertinent data from studies underscore that ankle sprains constitute 15% of injuries among collegiate athletes in the United States, with a notably elevated incidence rate among accomplished basketball players^6^. Initial investigations established that ankle sprains in numerous athletes often tend to recur^7^. In various team sports, the recurrence rate of ankle sprains in basketball approaches approximately 28%^8^, often attributed to neglect of rehabilitation following the initial injury^9,10^.

Chronic ankle instability (CAI) refers to a condition where, following one or more instances of ankle sprain, the ankle fails to fully recuperate, resulting in diminished stability of the ankle joint. This leads to recurrent episodes of ankle instability^11^. Such instability has the potential to detrimentally impact an athlete’s physical capabilities, performance in competitions, and daily activities. CAI patients commonly exhibit symptoms such as recurring sprains, altered sensory perceptions, impaired muscle control, swelling, pain, and reduced athletic prowess. As a prevalent ankle ailment, CAI is frequently observed among athletes, particularly in the realm of basketball^12^. This encompasses chronic lateral ankle sprains that compromise ankle joint stability and escalate the risk of in-game injuries for athletes^13^. CAI can lead to repetitive ankle sprains, consequently influencing an athlete’s performance in competitions and overall quality of life. CAI is caused by structural or functional instability of the ankle and peripheral soft tissues, changes in joint range of motion, abnormal balance function, and posture control in patients, with the main clinical manifestations being repeated ankle sprains^14^. Ankle sprains often result in laxity of the anterior talofibular ligament, with CAI patients showing a notable increase in the length of the anterior talofibular ligament^15^. In individuals with CAI, the affected ankle presents greater anterior displacement in the sagittal plane compared to healthy individuals, leading to atypical range of motion^16^. Football, basketball, and volleyball represent the sports most frequently associated with CAI incidence. Pertinent research underscores that approximately 70% of basketball players afflicted by acute ankle injuries encounter relapses within an 18-month span following the initial trauma, with nearly 20–40% of this subset progressing to develop CAI^17^. CAI, a prevalent disorder among basketball players, manifests with symptomatic ankle pain, muscular debility, proprioceptive deficiencies, and compromised functional performances; severe CAI detrimentally impinges upon specialized athletic capabilities^18^. The ramifications of CAI encompass diminished ankle muscle potency, restricted range of ankle motion, compromised proprioception, impaired self-reported functionality, hampered neuromuscular governance, and diminished equilibrium capabilities^19^. Moreover, CAI emerges as a pivotal contributor to lower limb structural injuries (such as anterior cruciate ligament, peroneal muscles, and superior peroneal retinaculum), and the interplay between structural and functional impairments of the lateral ankle drives the onset of CAI. This interrelationship is intertwined with neuromuscular and sensorimotor control aberrations^20^. Investigations have unveiled that individuals grappling with CAI exhibit decreased preactivation and a delay in the onset of peroneal muscle activation during dynamic tasks^21^. Researchers López-González et al. noted that patients with CAI exhibited postural stability deficits in both static and dynamic situations^22^.

The three-step layup is a common scoring technique in basketball games, encompassing the fundamental concept where, upon receiving the ball, the player executes a sequence of three consecutive steps to perform a layup shot. This technique aims to swiftly penetrate the defense and subsequently score. The three-step layup represents a basketball offensive maneuver involving three distinct stages: a short step, a long step, and a jump step. Employing this sequence of continuous footwork, players can rapidly approach the basket, evade defensive players, and create close-range scoring opportunities. Functioning as a scoring technique, the three-step layup not only enriches tactical choices within the game but also augments players’ prospects of scoring. Moreover, it serves as a test of players’ technical skills and decision-making abilities. In the realm of basketball, the execution of a three-step layup, encompassing a rapid single-leg jump (RSLJ) subsequent to a brief run-up, culminates in a landing on either one or both feet. During this intricate maneuver, the running pace is heightened, placing emphasis on the ball of the foot, and in certain instances, even the forefoot. This heightened pace engenders a dynamic interplay between the torso’s forward-moving inertia and the restraining force exerted by the lower limbs, consequently instigating a reciprocal action between body elevation and landing, rendering balance precarious. This interplay could lead to transient ankle joint inversion, precipitating the involvement of lateral ligaments, thereby predisposing to partial or even complete ligamentous rupture. Simultaneous rupture of the anterior talofibular and calcaneofibular ligaments may yield ankle joint instability, accompanied by temporary injury, dislocation, or subluxation^23^. Notably, the majority of ankle sprains occur upon landing on one or both feet^24,25^, thereby accentuating the significance of lower limb stability during landing to avert sports-related injuries. Within the domain of basketball, characterized by continuous takeoff and landing actions, the athlete’s airborne trajectory post-descent hinges on the takeoff foot’s strength and the direction of impact. The execution of a three-step layup demands players to swiftly perform consecutive footwork and jumping actions within a short timeframe, encompassing requisites pertaining to ankle stability, muscle control, and coordination^26^. However, individuals with CAI may encounter heightened biomechanical challenges during such maneuvers due to compromised ankle joint stability, potentially leading to instability in the execution of movements. The decreased stability of the ankle joint among CAI patients renders them more susceptible to injuries during physical activity, particularly in scenarios like the three-step layup that involve rapid movements and changes in direction, consequently elevating their risk of injury^27^. CAI could result in diminished control over the ankle joint for athletes, rendering them less capable of precise execution in actions requiring accurate footwork and jumping. The three-step layup necessitates the completion of multiple steps within a brief duration, placing substantial demands on ankle joint control and stability, a domain potentially affected by CAI. Due to functional loss and damage to ankle joints in CAI patients, when RSLJ is performed with the affected side as the starting foot, the height and landing stability of CAI patients will be affected to a certain extent^28^. However, the effects of CAI on the biomechanics of the ankle in the three-step layup of basketball players are still unclear, and many existing research results cannot be applied to basketball players. Therefore, this work focused on second-tier basketball players and aimed to investigate the impact of CAI on the biomechanical characteristics of ankle joints during the execution of a standard three-step layup maneuver. By analyzing the kinematic and kinetic parameter changes in the ankle joints of both CAI-afflicted and non-CAI basketball players, this work aimed to provide an in-depth understanding of how CAI influences the biomechanics of ankle joints during the three-step layup among basketball players. The results were intended to offer a foundation for informed choices in training methods for basketball players while also contributing valuable insights to the study of ankle-related conditions and the development of rehabilitation strategies.

## Materials and methods

### Study design

Prior to the commencement of testing, participants underwent a 3-min stationary warm-up session followed by a practice session involving the execution of a ball-less three-step layup. Subsequently, reflective marker points were meticulously affixed to predetermined anatomical locations on the lower limb surfaces of the subjects, meticulously adhering to the refinements incorporated in the Helen Hayes model^29^. The application of reflective markers was carried out by the same operator to ensure consistency in marker placement across different individuals. Since all the indicators required in this study were related to ankle joints, only marker points in the Helen Hayes model were selected to paste markers, which included the selection of landmarks on the ankle, knee center, hip joint center, thigh and shank, as well as the foot.

**Figure 1:**
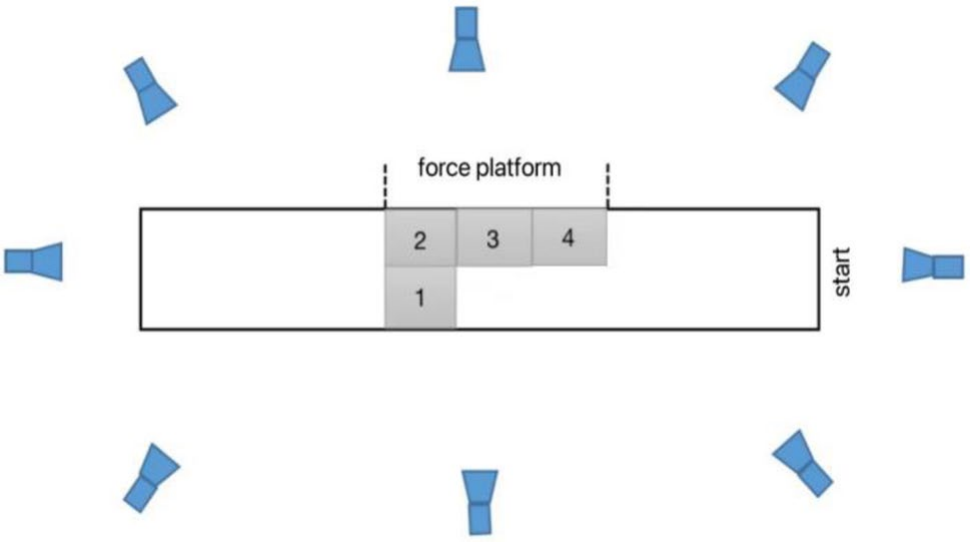
Camera position diagram.

In this study, all participants typically initiated the three-step layup maneuver using their left leg as the push-off support foot. Therefore, for the purpose of this study, the left leg should continue to serve as the push-off support foot. Following the approach run, the participants executed a ball-less three-step layup maneuver while adhering to a naturally inclined landing trajectory. The initial progression involved stepping onto the designated No. 4 force platform (Fig. 1) with the objective of vertically propelling the body into an aerial stance. Subsequently, they seamlessly transitioned to a natural landing posture—either on a single foot or both feet—proceeding to continue running post-landing on the designated No. 3 and No. 4 force platforms, respectively.

Definition of different phases in the three-step layup is as follows.

The one-foot push-off period refers to the moment during the execution of the three-step layup maneuver when one foot is off the ground, while the other foot is in contact with the floor. During this phase, the athlete utilizes the pushing force generated by the single supporting foot to lift off from the ground, preparing for the layup shot. This period typically occurs after the final step, where the player leverages the propulsion from the final step’s jump. The landing moment signifies the instance when an athlete completes the layup shot, and one foot makes contact with the ground after being airborne. At this moment, the player needs to land in a stable manner to prepare for subsequent actions, such as jumping again or changing direction. The stability during the landing moment is crucial to prevent injuries, ensuring the smooth execution and effectiveness of the subsequent actions. The one-foot landing period designates the duration between the time when one foot lands and the moment before the other foot lands in the three-step layup. Within this time frame, the athlete must maintain balance and control over their body posture to smoothly complete the maneuver. This phase typically involves flexion and control of the knee and ankle joints to ensure body stability.

### Participants and sample size calculation

Male second-level basketball players were selected and divided into a CAI group and a non-CAI group according to whether they were accompanied by CAI, with 15 participants in each group. Baseline data of athletes in the CAI and non-CAI groups are shown in Table 1. The qualifying criteria for CAI athletes were as follows^30^: (1) individuals meeting the criteria for CAI screening^31^; (2) unilateral ankle joint had at least one history of a severe sprain, pain, swelling, and other inflammatory symptoms within 2 years before the test; (3) unilateral ankle joint experienced uncontrollable and/or sprained and/or unstable sensation twice or more in the last 1 year; and (4) Cumberland Ankle Instability Tool (CAIT)^32^ score ≤ 27. Exclusion criteria were as follows: (1) athletes in the stage of acute injury and rehabilitation; (2) athletes unable to participate in normal classes, training, competition, and other activities for other reasons; and (3) athletes unwilling to participate in this experiment. Non-CAI inclusion criteria were as follows: normal basketball players without a history of ankle sprains, no head injury, acute lower limb injury, balance dysfunction, or chronic lower limb dysfunction within the past 3 months, and CAIT score > 27 points. The experimental procedure of this work was approved by the Ethics Committee of the Sports Science Experiment of Beijing Sport University (No. 2022154H), and all subjects signed informed consent forms. During the work process, all methods were performed in accordance with the relevant guidelines and regulations.

**Table 1:** Participant demographics.

Group	Age (years old)	Height (cm)	Weight (kg)	CAIT scores
CAI	21.29 ± 2.63	188.86 ± 7.13	88.43 ± 5.26	15.29 ± 3.15
Non-CAI	20.43 ± 1.81	186.57 ± 6.99	88.00 ± 6.93	28.71 ± 1.11

The sample size was calculated using GPower 3.0.18, with the three-step layup ankle biomechanics as the main result. This study was anticipated to encompass four measurement variables in both the CAI group and the non-CAI group. The effect size is employed as an estimation of the standard mean difference—a metric that is independent of the current sample and quantifies the magnitude of differences between distinct sampled populations. Effect size provides a more accurate description of differences. It signifies the degree of overlap between two population distributions; larger effect sizes indicate lesser overlap and more pronounced differences, while smaller effect sizes indicate the opposite. Variance analysis effect sizes are categorized as 0.1 (small effect), 0.3 (medium effect), and 0.5 (large effect)^33^. Hence, to comprehensively and objectively analyze varying conditions and actions, the effect size was set at 0.5. This adjustment reduces the overlap among the population distributions, enhancing the clarity of differences. A statistical significance level (α) of 0.02 and a statistical power (1-β) of 0.8 were established. The calculated total sample size was determined to be 12 individuals. Ultimately, this study included a total of 30 participants.

### Data collection

In this study, four Kistler 9281CA (Switzerland) 3D force measuring tables (specification: 40 cm × 60 cm × 10 cm) were used to collect and process the subjects’ movements. The Motion Analysis Raptor-4 high-speed infrared motion capture system, equipped with 8 cameras, along with a force plate from Motion Analysis company, was employed to collect kinematic parameters of reflective markers on the subjects. The camera system features a resolution of 2352 × 1728 pixels and operates at a sampling frequency of 200 Hz. Data collection was conducted over a period of 10 s. The collection of kinematic and dynamic data was triggered synchronously. To ensure the validity of the experimental data, each subject collected valid data 3 times.

Prior to testing, spatial calibration was conducted to ensure that subjects were entirely captured after completing their actions and to prevent interference from other reflective objects that might affect data collection. The general area of subject movement was defined within the testing environment, and calibration was carried out using a Direct Linear Transformation (DLT) technique. Personnel sequentially calibrated the determined area using calibration poles, guaranteeing comprehensive spatial coverage. The calibration space encompassed the range above the testing area, measuring 3.0 m × 2.5 m × 2.5 m, corresponding to the region where subjects performed the test actions.

The variable selected in this study were ankle angle, moment, and ground reaction force^34^. In data processing, the link coordinate system was established through infrared reflective marks on the body surface of the subject, and the moment and angle of the ankle joint were calculated according to the trajectory change of the movement and the coordinate system. The index definition of the left ankle was as follows: in ankle angle and moment, the ankle joint performs dorsiflexion and plantarflexion movements in the sagittal plane, where positive values represent plantarflexion and negative values represent dorsiflexion. It also executes inversion and eversion movements in the frontal plane, with positive values denoting inversion and negative values denoting eversion. Additionally, internal and external rotation movements occur in the transverse plane, where positive values signify internal rotation and negative values signify external rotation. Utilizing the Helen Hayes human body model parameters, 3D moments of force at the ankle joint were computed using inverse dynamics method^35^. Kinematic and kinetic data were concurrently collected using the infrared motion capture system, Seeker.

### Data processing

Cortex (version 2.6.2.1169) was used to process the kinematics data, and Kistler Bio Ware (version 3.2.6.104) was used to extract the dynamics data. The Butterworth low-pass filtering method was used to smooth the 3d coordinates of all identification points, and the truncation frequency was 10 Hz^36^.

The center of the knee joint is located at the midpoint between the medial and lateral knee points, while the center of the ankle joint is situated at the midpoint between the medial and lateral ankle points. Once the ankle joint center position was determined, a ground coordinate system (GCS) was established using corresponding marker points. Additionally, a local coordinate system (LCS) for the ankle joint was established. For the knee joint, the origin of the coordinate system was set at the knee joint center, the line connecting the right and left points defined the positive X-axis direction, and the line from the distal end to the proximal end established the Z-axis. The Y-axis was then derived by computing the cross product of the normal vectors of the X-axis and Z-axis. For the ankle joint coordinate system, the X-axis aligned with the lower leg, the Z-axis pointed from the ankle joint center to the tip of the foot, and similarly, the Y-axis of the foot coordinate system was obtained through a cross product.

In accordance with the original force plate setup and the configuration of the testing environment, the Z-axis of the force plate was oriented vertically upwards, the X-axis corresponded to the direction of a sudden stop jump shot’s jumping motion, and the Y-axis extended from the athlete’s right to left side. In the Cortex software, the pressure threshold of the force plate was adjusted to 10 N, enabling the automatic exclusion of force values below 10 N. Force plate data was exported from the Cortex, and using the Helen Hayes human body model parameters, inverse dynamics were applied to compute the magnitude of the 3D ankle joint moments of force. The ankle joint’s 3D moments of force were calculated based on the Winter formula.

### Statistical analysis

In this research, the calculation of force and moment variables was normalized by weight. Additionally, relative value comparison was employed to assess differences among the various data sets. First, the normality of the data was examined using the Shapiro–Wilk test, while the homogeneity of the data was analyzed using the Bartlett test. The significance criterion was set at a type I error probability not exceeding 0.05. The analysis results indicated that all data in this study adhered to a normal distribution and met the assumption of homogeneity of variance. Statistical parametric mapping (SPM) was adopted for the test. The peak values of the maximum foot flexion angle, the maximum foot inversion angle, the maximum dorsiflexion angle, and the maximum ankle dorsiflexion moment in the two groups of subjects were analyzed by using the paired sample *t* test, and the statistical significance level was set to *P* < 0.05. *T* tests and SPM were performed using SPSS 22.0 (IBM, USA) and MATLAB R2016a (MathWorks, USA), respectively.

## Result

### One-foot push-off period

**Figure 2:**
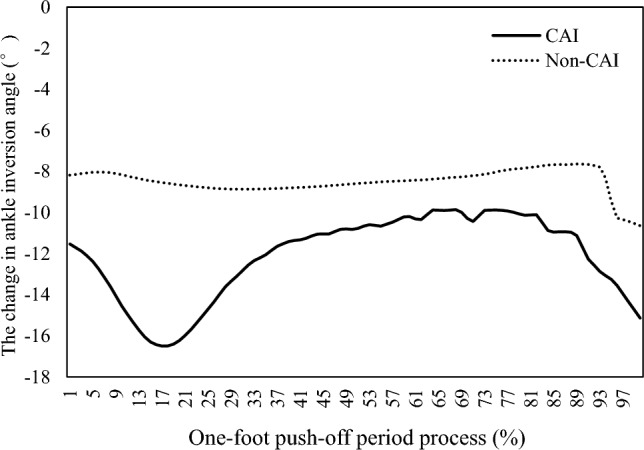
The relationship between ankle inversion angle and time percentage during the one-foot push-off phase.

In Table 2, the maximum plantar flexion angle of the ankle was 3.63 ± 1.62° in the CAI group and 3.45 ± 1.22° in the non-CAI group, and there was no significant difference between the two groups (t = 0.351, *P* > 0.05). The maximum ankle joint inversion angles in the CAI and non-CAI groups were 11.82 ± 5.05° and 16.52 ± 3.32°, respectively. The maximum ankle joint inversion angle in the CAI group was significantly smaller than that in the non-CAI group, and the difference between the two groups was extremely significant (t = − 3.006, *P* < 0.01). Therefore, there was no significant difference in the peak time of the maximum plantar flexion angle and maximum inversion angle of the ankle between the CAI group and the non-CAI group (*P* > 0.05). Figure 2 also shows that during the one-foot push-off period, the ankle inversion angle in the CAI group changed less than that in the non-CAI group.

**Table 2:** Ankle angle during takeoff on one foot.

Maximum angle of ankle joint	CAI (n = 15)	Non-CAI (n = 15)	t	P
Inversion (°)	11.82 ± 5.05	16.52 ± 3.32	− 3.006	0.006**
peak time (s)	0.08 ± 0.09	0.05 ± 0.02	1.054	0.329
Plantarflexion (°)	3.63 ± 1.62	3.45 ± 1.22	0.351	0.728
peak time (s)	0.14 ± 0.05	0.11 ± 0.05	1.254	0.234

**Significance was accepted for *P* < 0.01.

The peak moment in this part refers to the maximum ankle moment in the whole stage of the one-foot push-off. When taking off, the lower limbs exert force, and the ankle joints move in the direction of plantar flexion. The following results can be observed from Table 3. The mean value of the peak plantar-flexor moment of the CAI group was (4.20 ± 1.06) N m, while that of the non-CAI group. was (5.07 ± 2.74) N·m. The difference between the two groups was not statistically significant (t = − 0.781, *P* > 0.05), and there was no significant difference in the occurrence time of peak moment between the two groups (t = − 0.148, *P* > 0.05).

**Table 3:** Peak torque during push-off on one foot.

Peak torque	CAI (n = 15)	Non-CAI (n = 15)	t	*P*
Plantarflexion (N m)	4.20 ± 1.06	5.07 ± 2.74	− 0.781	0.45
peak time (s)	0.13 ± 0.05	0.13 ± 0.04	− 0.148	0.885

### Landing moment

In Table 4, there were no statistically significant differences in ankle inversion angle and ankle dorsiflexion angle between the CAI group and the non-CAI group on landing (*P* > 0.05).

**Table 4:** Ankle angle at landing.

Ankle angle	CAI (n = 15)	Non-CAI (n = 15)	t	*P*
Inversion (°)	15.52 ± 5.38	14.24 ± 5.58	0.638	0.528
Dorsiflexion (°)	34.67 ± 11.37	37.61 ± 14.34	− 0.620	0.541

The concept of 3D moment encompasses the magnitude of the ankle joint moment across three distinct orientations: the sagittal plane, oriented vertically; the frontal plane, spanning the lateral axis; and the horizontal plane, encompassing the anteroposterior axis. During the phases of takeoff and landing, the lateral force magnitude denotes the ankle joint’s lateral stability, while the anterior–posterior force magnitude signifies forward acceleration or deceleration. As delineated in Table 5, no statistically significant variance emerged in ankle torque across the sagittal, frontal, and horizontal planes between the CAI and non-CAI groups (*P* > 0.05).

**Table 5:** Three-dimensional moment of the ankle joint at landing.

Three dimensional	CAI (n = 15)	Non-CAI (n = 15)	t	*P*
Sagittal plane (N m)	0.18 ± 0.18	0.11 ± 0.01	0.928	0.372
Frontal plane (N m)	0.01 ± 0.03	1.31 ± 2.59	− 1.323	0.234
Horizontal plane (N m)	0.02 ± 0.03	0.01 ± 0.01	1.232	0.241

### One foot landing period

In Table 6, during the falling and landing of the three-step layup, the maximum inversion angle was (18.15 ± 4.66)° in the CAI group and (9.98 ± 2.83)° in the non-CAI group. The maximum inversion angle was significantly larger in the CAI group than in the non-CAI group (t = 8.802, *P* < 0.001). The maximum dorsiflexion angle of the ankle joint in the CAI group was 11.32 ± 5.73°, and that in the non-CAI group was 7.28 ± 3.83°. The maximum dorsiflexion angle of the ankle joint in the CAI group was significantly larger than that in the non-CAI group (t = 2.265, *P* < 0.05). However, there was no significant difference in the peak time of the maximum inversion angle and maximum dorsiflexion angle of the ankle between the CAI and non-CAI groups (*P* > 0.05).

**Table 6:** Ankle angle during the one-foot landing period.

Maximum angle of ankle joint	CAI (n = 15)	Non-CAI (n = 15)	t	*P*
Inversion (°)	18.15 ± 4.66	9.98 ± 2.83	8.802	< 0.001***
Peak time (s)	0.03 ± 0.04	0.05 ± 0.10	− 0.654	0.526
Dorsiflexion (°)	11.32 ± 5.73	7.28 ± 3.83	2.265	0.031*
Peak time (s)	0.12 ± 0.04	0.10 ± 0.03	1.197	0.254

*Significance was accepted for *P* < 0.05, ***significance was accepted for *P* < 0.001.

**Figure 3:**
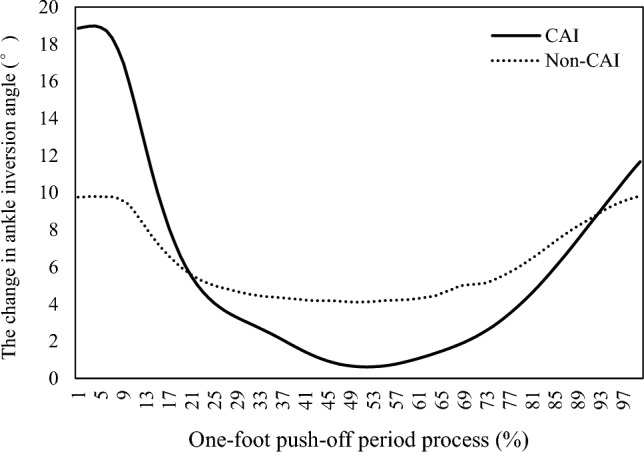
The correlation between ankle inversion angle and time percentage during the one-foot push-off phase.

In the whole landing stage, the change in ankle inversion angle in the CAI group was significantly greater than that in the non-CAI group (Fig. 3), indicating that the range of ankle inversion motion in the basketball players with CAI was larger than that in the athletes with a stable ankle in the non-CAI group.

In Table 7, the peak dorsiflexion moment of the ankle joint in the CAI group and the non-CAI group was (2.39 ± 0.52) N m and (2.78 ± 0.85) N·m in the process of landing on one foot, but there was no significant difference between the two groups (t = 1.053, *P* > 0.05). The peak ankle dorsiflexion moment was 0.15 ± 0.05 s in the CAI group and 0.08 ± 0.06 s in the non-CAI group. Therefore, the peak time of ankle dorsiflexion moment in the CAI group was longer than that in the non-CAI group (t = − 2.428, *P* < 0.05).

**Table 7:** Peak torque during landing on one foot.

Peak torque	CAI (n = 15)	Non-CAI (n = 15)	t	*P*
Dorsiflexion (N m)	2.39 ± 0.52	2.78 ± 0.85	1.053	0.313
peak time (s)	0.15 ± 0.05	0.08 ± 0.06	− 2.428	0.032*

*Significance was accepted for *P* < 0.05.

## Discussion

While previous investigations have typically segregated takeoff and landing as distinct entities, a contrasting perspective has been proposed by Akbari et al.^37^, positing that the “jump-land” sequence constitutes an integrated sequence of actions. This perspective underscores the interdependence between the takeoff and landing components, particularly the strategic considerations governing the jumping phase, in the context of movements like the three-step layup’s RSLJ pattern. This pattern culminates in landing on either a single foot or both feet, serving as a foundational scoring technique and a fundamental basketball maneuver. The stability achieved during the post-push-off landing phase assumes a pivotal role in safeguarding athletes against sports-related injuries. Notably, ground landing diverges from takeoff and entails varied foot positioning contingent on individual differences. Therefore, this study embraces the holistic concept of “jump-land” as an integrated entity and scrutinizes the kinematic attributes of ankle instability during basketball players’ layup sequences at distinct time intervals. By conducting a comprehensive analysis of ankle angle, torque, and related variables across various directions between the experimental and control groups, this study examined the foot kinematic attributes of basketball players afflicted by CAI. Additionally, the impact of ankle stability on fundamental basketball techniques and overall sports performance was investigated. The outcomes of this investigation revealed a noteworthy decrease in the maximum ankle joint inversion angle during the one-foot push-off phase among individuals with CAI. Conversely, the one-foot landing phase showcased an elevation in both the maximum inversion angle and maximum dorsiflexion angle of the ankle joint. Furthermore, the peak time of ankle dorsiflexion moment exhibited an extended duration in individuals with CAI.

Angular characteristics of the ankle joint across distinct phases of the three-step layup hold significant scholarly interest. Prior research, both domestic and international, has predominantly concentrated on dissecting and explicating the kinematic and mechanical attributes of the lower extremities, encompassing the hip, knee, and ankle joints. Such studies have formulated optimized movement patterns for diverse actions through a thorough examination of pertinent indicators^38^. However, there remains a dearth of inquiries into the impact of CAI on the ankle joint biomechanics during basketball players’ execution of the three-step layup. Notably, a subset of researchers has ascertained that the muscles governing ankle joint dorsiflexion and plantar flexion predominantly assume the role of prime motor units during the pedal extension phase of the push-off leg. In this context, the muscles controlling plantar flexion movements manifest the highest activation levels. Consequently, the primary motive force in the ankle joint during this juncture emanates from plantar flexion^39^. Ankle joint injuries among basketball players frequently result in an inversion disposition. Consequently, the lateral malleolus ligaments experience a gradual reduction in both elasticity and tautness due to injury. Within the ankle joint, the talofibular and calcaneal fibular ligaments operate synergistically to counteract ankle inversion. Particularly, the anterior talofibular ligament displays heightened tension during plantar flexion, serving the purpose of curbing excessive inversion during this phase. Conversely, the calcaneal fibular ligament attains its maximal tension during dorsiflexion, functioning to deter ankle pronation in the dorsiflexion plane. The results of this study showed that the maximum ankle inversion angle in the CAI group was significantly smaller than that in the non-CAI group. This phenomenon contrasts with the maximal angle shift of the ankle joint in cases of CAI incidence. A heightened ankle joint angle during takeoff signifies an advantageous augmentation of initial velocity. Conversely, an excessively diminished angle may accentuate resistance from the knee flexor musculature. Moreover, the ankle muscle robustness and suppleness in CAI-afflicted individuals exert an influence on ankle joint extension angles. Conversely, CAI participants demonstrated a greater range of ankle joint motion during abrupt halts^40^. However, in the CAI group, the maximal peak value and range of inversion angle were comparatively smaller than those observed in the non-CAI group, a divergence potentially linked to dissimilar modes of movement. In alignment with Cho et al.^41^, our study resonates with the notion that the atrophy of ankle joint-supporting musculature significantly contributes to the waning physical performance in CAI patients. Moreover, compromised postural control may be intertwined with limitations in ankle joint mobility. Consequently, the outcomes of this research possibly stem from biomechanical shifts within ankle joints among CAI patients, curbing both lower limb ankle strength and the range of joint motion during takeoff^42^.

Landing constitutes a ubiquitous motion in both daily life and sports, characterized by an initial touchdown on the forefoot, followed by swift transition to the sole. This action is accompanied by concurrent hip flexion, knee bending, and ankle extension. Scholars have emphasized that augmenting the range of motion in ankle joint plantar flexion and dorsiflexion during the landing phase can enhance the ankle joint’s shock-absorbing capacity^43^. Allet et al.^44^ conducted an assessment of the ankle angle among CAI patients pre- and post-landing, concluding that patients with subacute ankle sprains displayed larger dorsiflexion angles both before and after ground contact compared to non-CAI individuals. Similarly, Kim et al.^45^ observed that, during initial ankle joint-ground contact, individuals with ankle instability exhibited a reduced rearfoot plantar flexion angle in contrast to both the Coper group (those with potential for CAI) and the control group. Furthermore, a noticeable trend of increasing dorsiflexion angle was noted. This phenomenon is attributed to the proclivity of CAI patients to experience episodes of “giving away” (ankle instability) during daily activities and sports. This instability, stemming from damage to the talofibular ligament, engenders ligamentous relaxation relative to non-CAI counterparts. Consequently, the dorsiflexed position of the ankle joint during descent affords heightened stability. Dorsiflexion also effectively mitigates undue stress on the lateral ligaments of the ankle. The reflexive self-protective mechanism exhibited by patients prompts an innate adjustment to augment dorsiflexion angle, thereby mitigating the risk of injury.

From the analysis of the whole landing movement process, the push-off height of athletes when they perform the three-step layup usually exceeds 50% of their height. The higher the height is, the greater the impact of the ground on the feet. In this study, the ankle inversion angle of subjects in the CAI group was higher than that in the non-CAI group when landing, but there was no significant difference in the ankle inversion angle and ankle dorsi flexion angle between the CAI group and non-CAI group when landing (*P* > 0.05). The findings of this study elucidate a correlation between alterations in ankle inversion angle and changes in joint muscle strength. Dynamic stability of the ankle joint hinges on the coordinated contraction of the surrounding muscle groups. In various lower limb activities like walking, running, and jumping, athletes rely on concerted muscle contractions, particularly centrifugal contractions, to regulate the interplay of forces between the foot and the ground. Joint injury triggers peripheral neuromuscular perturbations, precipitating a continuous reflexive response in the muscles. For the preservation of bodily posture stability, individuals with CAI must curtail the range of motion in hip and knee joints. This action serves to mitigate the lateral swaying of the torso upon unilateral landing of the ankle joint. Nonetheless, due to the substantial ground reaction force encountered during early ground contact, the ankle joint necessitates a notable range of motion to ensure landing stability.

Torque profiles of the ankle across distinct stages of the three-step layup exhibit the subsequent characteristics. Notably, it has been observed that the peak plantar flexor moment of the ankle joint in CAI patients registers a significant reduction compared to that in individuals without ankle instability. This phenomenon signifies inadequate function within the plantar flexor muscle group during natural ambulation^46^. Further investigations reveal that the peak plantar-flexion moment in CAI patients, across various angular velocities, remains consistently lower than in their healthy counterparts. These insights underscore functional impairments in plantar-flexion capabilities among CAI patients, particularly evident during routine and sport-specific activities, rendering them susceptible to recurrent ankle sprains. Comparative analyses of peak plantar-flexion moment values at different temporal intervals between the two cohorts unveil a salient finding. Specifically, this study discerns that during single-leg takeoff, the experimental group of CAI subjects exhibits diminished peak plantar-flexion moment values in contrast to their normally functioning counterparts in the control group. In the context of the three-step layup technique, athletes engage in a running approach, followed by a forceful leap that propels them both forward and upward. As they ascend, they aim to attain the highest point in their trajectory precisely as the ball enters the basket. This strategy maximizes the effectiveness of the jump shot, with proximity to the basket correlating to an elevated leap. Consequently, athletes must not only possess robust lower limb explosive strength but also exhibit proficient control over anterior–posterior directionality in the ankle. Given that the ankle joint’s movement involves the interplay of plantar and dorsiflexion during takeoff, and joint torque is intricately influenced by muscle strength and stability, empirical investigations have confirmed that individuals with CAI exhibit inadequate ankle joint muscle strength and diminished postural control capacity. Thus, the ankle joint serves as the pivotal point for initiating lower limb joint forces during the takeoff phase of the three-step layup. In CAI patients, deficient muscle strength within the ankle joint’s plantar and dorsiflexor muscles contributes to a weakened ability to generate upward propulsive forces against the ground and to propel the lower limbs forward. This dynamic ultimately exerts a detrimental impact on sports performance.

The moment of the ankle joint in the frontal plane of the CAI group was smaller than that in the control group. When the ankle joint touched the ground for the first time, the ankle joint of the CAI group showed a decrease in the function of inversion and inversion muscle compared with the normal person, while the moment of the sagittal plane and horizontal plane showed no significant difference. This may be related to the fact that the toe just touched the ground at the moment of touching the ground and had not yet been subjected to a large ground reaction force, so there was no difference between the two groups in the sagittal plane and horizontal plane. On the other hand, it may also be related to the large pronation angle of CAI at the moment of touching the ground. Due to insufficient muscle strength, it is difficult for the ankle joint to control and land safely within a stable range when falling to the ground, which increases the risk of ankle injury or CAI when landing. As the plantar flexors all touch the ground in the landing process, the comparison of the peak torque in the plantar flexor direction shows that the CAI result is smaller than that of the non-CAI group. Although the difference is not obvious, the arrival time of the peak torque in the CAI group is delayed. Both Wang^47^ and Thompson^48^ found through the isokinetic muscle force test that the peak values of eversion and plantar-flexion moment of patients with CAI at different angular speeds were smaller than those of healthy people. The results were consistent with this study, but the difference was that the isokinetic muscle force test obtained the peak values of ankle moment by setting a fixed angular velocity and movement direction. In this study, the peak value of ankle moment was collected when subjects fell naturally, without setting a uniform speed and direction. In this case, it is more consistent with the actual situation of athletes in the process of sports, which has practical significance for later training practice and injury prevention.

However, there were still some shortcomings in this work. It was pointed out that the test subjects in this work all run at the same starting way and speed, which was difficult to do in the training field. Therefore, in future work, the influence of CAI on the dynamics of basketball players’ ankle joints under different takeoff modes and running speeds will be further analyzed.

## Conclusion

This work discussed the changes in ankle biomechanical indexes of CAI and non-CAI basketball players in typical three-step layup movement from the perspectives of dynamics and kinematics. The results showed that the maximum ankle joint inversion angle decreased during the one-foot push-off period of CAI. The maximum inversion angle and maximum dorsiflexion angle of the ankle joint increased during the one-foot landing period, and the peak time of ankle dorsiflexion moment was prolonged. In conclusion, this work provided a reference for the choice of training methods for basketball players with CAI.

### Supplementary Information

Supplementary Information 1.

Supplementary Information 2.

Supplementary Information 3.

Supplementary Information 4.

## Data Availability

All data generated or analysed during this study are included in this published article [and its supplementary information files].

## References

[1] Padua E, D'Amico AG, Alashram A (2019). Effectiveness of warm-up routine on the ankle injuries prevention in young female basketball players: A randomized controlled trial. Medicina.

[2] Sheppard J, Gabbett T, Stanganelli LCR (2010). An analysis of playing positions in elite international mens' volleyball: Considerations for competition demands and physiological characteristics. J. Strength Cond. Res..

[3] Sheppard JM, Gabbett TJ, Stanganelli LC (2009). An analysis of playing positions in elite men's volleyball: considerations for competition demands and physiologic characteristics. J. Strength Cond. Res..

[4] Panagiotakis E, Mok KM, Fong DT, Bull AMJ (2017). Biomechanical analysis of ankle ligamentous sprain injury cases from televised basketball games: Understanding when, how and why ligament failure occurs. J. Sci. Med. Sport..

[5] Silva JR, Rumpf MC, Hertzog M (2018). Acute and residual soccer match-related fatigue: A systematic review and meta-analysis. Sports Med..

[6] Pasanen K, Ekola T, Vasankari T (2017). High ankle injury rate in adolescent basketball: A 3-year prospective follow-up study. Scand. J. Med. Sci. Sports.

[7] Tummala SV, Hartigan DE, Makovicka JL (2018). 10-year epidemiology of ankle injuries in men’s and women’s collegiate basketball. Orthop. J. Sports Med..

[8] Roos KG, Kerr ZY, Mauntel TC (2017). The epidemiology of lateral ligament complex ankle sprains in national collegiate athletic association sports[J]. Am. J. Sports Med..

[9] Yin Y, Yu Z, Wang J, Sun J (2022). Effectiveness of the rehabilitation training combined with maitland mobilization for the treatment of chronic ankle instability: A randomized controlled trial. Int. J. Environ. Res. Public Health.

[10] Hunt KJ, Hurwit D, Robell K, Gatewood C, Botser IB, Matheson G (2017). Incidence and epidemiology of foot and ankle injuries in elite collegiate athletes. Am. J. Sports Med..

[11] Lian J, Sewani F, Dayan I (2022). Systematic review of injuries in the men's and women's national basketball association. Am. J. Sports Med..

[12] Attenborough AS, Hiller CE, Smith RM, Stuelcken M, Greene A, Sinclair PJ (2014). Chronic ankle instability in sporting populations. Sports Med..

[13] Toyooka T, Urabe Y, Sugiura S, Takata A, Shinozaki M, Takata Y, Ishizaki T, Nakamura K, Otsuki K, Oyama T, Nishikawa S (2018). Does the single-limb stance reflect chronic ankle instability in an athlete?. Gait. Posture.

[14] Hertel J, Corbett RO (2019). An updated model of chronic ankle instability. J. Athl. Train..

[15] Herzog MM, Kerr ZY, Marshall SW, Wikstrom EA (2019). Epidemiology of ankle sprains and chronic ankle instability. J. Athl. Train..

[16] Hall EA, Chomistek AK, Kingma JJ, Docherty CL (2018). Balance- and strength-training protocols to improve chronic ankle instability deficits, part I: Assessing clinical outcome measures. J. Athl. Train..

[17] Biz C, Nicoletti P, Tomasin M, Bragazzi NL, Di Rubbo G, Ruggieri P (2022). Is kinesio taping effective for sport performance and ankle function of athletes with chronic ankle instability (CAI)? A systematic review and meta-analysis. Medicina.

[18] Miklovic TM, Donovan L, Protzuk OA, Kang MS, Feger MA (2018). Acute lateral ankle sprain to chronic ankle instability: A pathway of dysfunction. Phys. Sportsmed..

[19] Cain MS, Ban RJ, Chen YP, Geil MD, Goerger BM, Linens SW (2020). Four-week ankle-rehabilitation programs in adolescent athletes with chronic ankle instability. J. Athl. Train..

[20] Delahunt E, Remus A (2019). Risk factors for lateral ankle sprains and chronic ankle instability. J. Athl. Train..

[21] Picot B, Hardy A, Terrier R, Tassignon B, Lopes R, Fourchet F (2022). Which functional tests and self-reported questionnaires can help clinicians make valid return to sport decisions in patients with chronic ankle instability? A narrative review and expert opinion. Front. Sports Act Living.

[22] López-González L, Falla D, Lázaro-Navas I (2021). Effects of dry needling on neuromuscular control of ankle stabilizer muscles and center of pressure displacement in basketball players with chronic ankle instability: A single-blinded randomized controlled trial. Int. J. Environ. Res. Public Health.

[23] Al Adal S, Pourkazemi F, Mackey M, Hiller CE (2019). The prevalence of pain in people with chronic ankle instability: A systematic review. J. Athl. Train..

[24] Guo J, Yang J, Wang Y, Mo Z, Pu J, Fan Y (2022). Effect of different protection on lateral ankle during landing: An instantaneous impact analysis. Bioengineering.

[25] Choi IR, Lee JH (2021). Effects of shoes that can be tightened using wire and dial on the dynamic balance following ankle muscle fatigue: A crossover study. Healthcare.

[26] Shiravi Z, Shadmehr A, Moghadam ST, Moghadam BA (2017). Comparison of dynamic postural stability scores between athletes with and without chronic ankle instability during lateral jump landing. Muscles Ligaments Tendons J..

[27] Emamvirdi M, Hosseinzadeh M, Letafatkar A, Thomas AC, Dos'Santos T, Smania N, Rossettini G (2023). Comparing kinematic asymmetry and lateral step-down test scores in healthy, chronic ankle instability, and patellofemoral pain syndrome female basketball players: A cross-sectional study. Sci. Rep..

[28] Lin CI, Mayer F, Wippert PM (2022). The prevalence of chronic ankle instability in basketball athletes: A cross-sectional study. BMC Sports Sci. Med. Rehabil..

[29] Zhang C, Chen N, Wang J (2022). The prevalence and characteristics of chronic ankle instability in elite athletes of different sports: A cross-sectional study. J. Clin. Med..

[30] Morikawa LH, Tummala SV, Brinkman JC, Buckner Petty SA, Chhabra A (2022). Effect of a condensed NBA season on injury risk: An analysis of the 2020 season and player safety. Orthop. J. Sports Med..

[31] Nedelec M, Dupont G (2019). The influence of playing position in soccer on the recovery kinetics of cognitive and physical performance. J. Sports Med. Phys. Fitness.

[32] Khan B, Ikram M, Rehman SSU, Un NZ (2022). Urdu translation and cross-cultural validation of cumberland ankle instability tool (CAIT). BMC Musculoskelet. Disord..

[33] Vella SP, Swain M, Downie A, Howarth SJ, Funabashi M, Engel RM (2023). Induced leg length inequality affects pelvis orientation during upright standing immediately following a sit-to-stand transfer: A pre-post measurement study. BMC Musculoskelet. Disord..

[34] Ji Y, Xu R, Zuo H, Wang Z, Jin H (2021). Biomechanics analysis of the lower limbs in 20 male sprinters using the international society of biomechanics six-degrees-of-freedom model and the conventional gait model. Med. Sci. Monit..

[35] Northeast L, Gautrey CN, Bottoms L, Hughes G, Mitchell ACS, Greenhalgh A (2018). Full gait cycle analysis of lower limb and trunk kinematics and muscle activations during walking in participants with and without ankle instability. Gait Posture.

[36] Yang C, Yao W, Garrett WE (2018). Effects of an intervention program on lower extremity biomechanics in stop-jump and side-cutting tasks. Am. J. Sports Med..

[37] Akbari H, Shimokochi Y, Sheikhi B (2023). Ankle dorsiflexion range of motion and landing postures during a soccer-specific task. PLoS One.

[38] Deng L, Yang Y, Yang C (2021). Compression garments reduce soft tissue vibrations and muscle activations during drop jumps: An accelerometry evaluation. Sensors.

[39] Terada M, Pietrosimone BG, Gribble PA (2014). Alterations in neuromuscular control at the knee in individuals with chronic ankle instability. J. Athl. Train..

[40] Wang B, Zhang X, Zhu F (2022). A randomized controlled trial comparing rehabilitation with isokinetic exercises and Thera-Band strength training in patients with functional ankle instability. PLoS One.

[41] Cho BK, Park JK, Choi SM, Kang SW, SooHoo NF (2019). The peroneal strength deficits in patients with chronic ankle instability compared to ankle sprain copers and normal individuals. Foot Ankle Surg..

[42] Kobayashi T, Watanabe K, Ito T (2019). The effect of novel ankle-realigning socks on dynamic postural stability in individuals with chronic ankle instability. Int. J. Sports Phys. Ther..

[43] Lee J, Song Y, Shin CS (2018). Effect of the sagittal ankle angle at initial contact on energy dissipation in the lower extremity joints during a single-leg landing. Gait Posture.

[44] Allet L, Zumstein F, Eichelberger P, Armand S, Punt IM (2017). Neuromuscular control mechanisms during single-leg jump landing in subacute ankle sprain patients: A case control study. PM R..

[45] Kim H, Son SJ, Seeley MK, Hopkins JT (2019). Altered movement biomechanics in chronic ankle instability, coper, and control groups: Energy absorption and distribution implications [published correction appears in J Athl Train. 2020 Jan; 55(1)5]. J. Athl. Train.

[46] Gross, C. E., Goodloe, J. B., Nunley, J. A. Management of Chronic Ankle Instability in the Basketball Player. *Basketb. Sports Med. Sci.*, 459–466 (2020).

[47] Wang J, Zhang D, Zhao T, Ma J, Jin S (2021). Effectiveness of balance training in patients with chronic ankle instability: Protocol for a systematic review and meta-analysis. BMJ Open.

[48] Thompson C, Schabrun S, Romero R, Bialocerkowski A, van Dieen J, Marshall P (2018). Factors contributing to chronic ankle instability: A systematic review and meta-analysis of systematic reviews. Sports Med..

