# Supplementary figures and images for: Ankle biomechanics of the three-step layup in a basketball player with chronic ankle instability
Source: Sci Rep. 2023 Oct 31;13:18667. doi: 10.1038/s41598-023-45794-w (PMC10618240; doi:10.1038/s41598-023-45794-w)

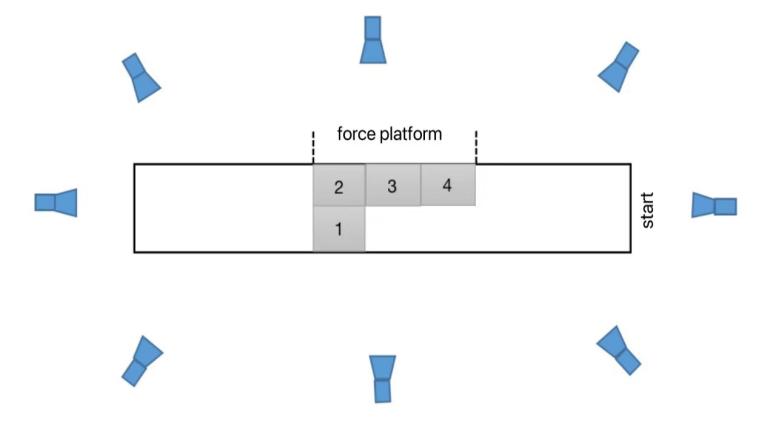

Supplement: Supplementary file 2 — Supplementary Information 2. [file 41598_2023_45794_MOESM2_ESM.jpg]

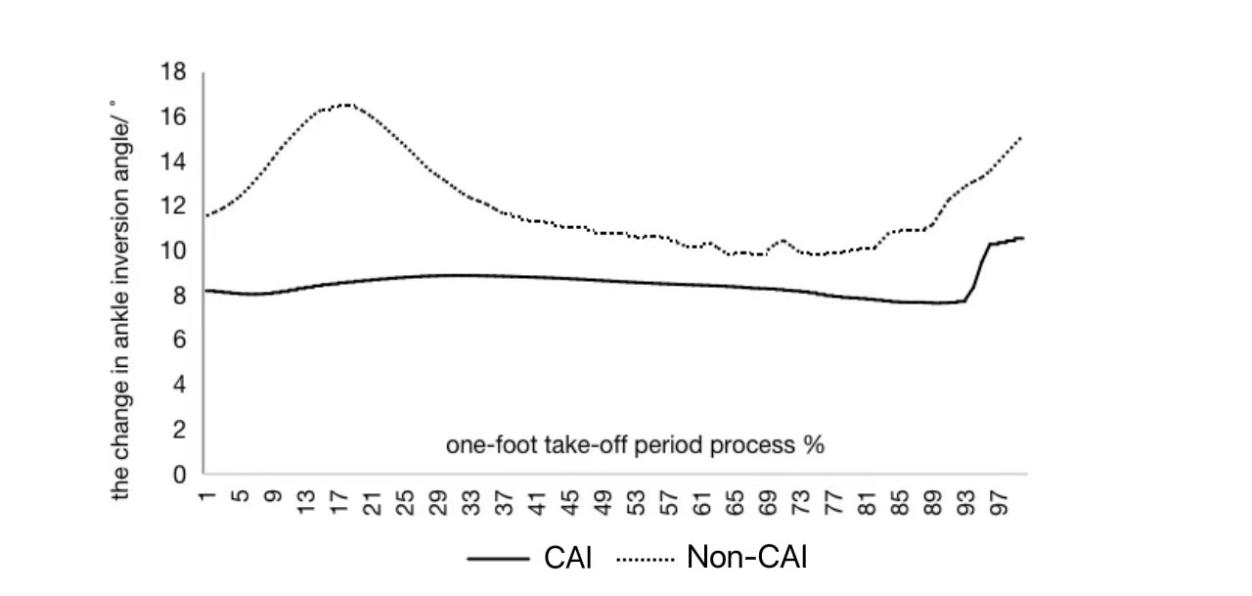

Supplement: Supplementary file 3 — Supplementary Information 3. [file 41598_2023_45794_MOESM3_ESM.jpg]

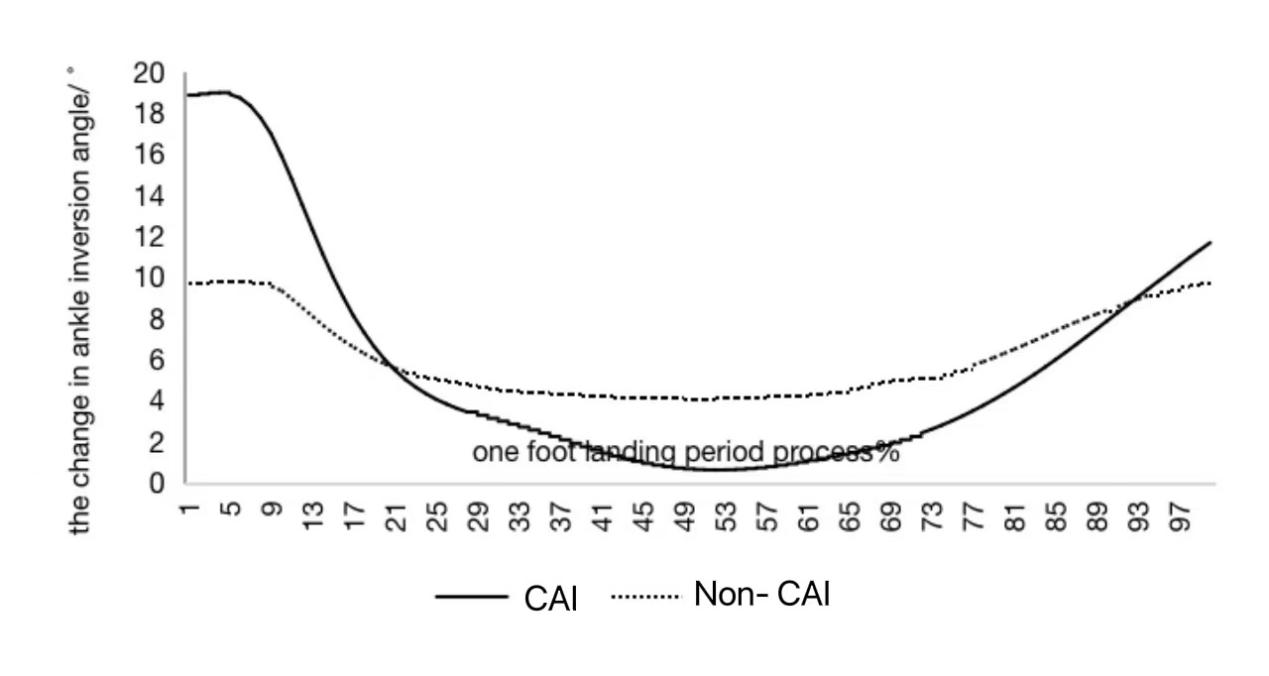

Supplement: Supplementary file 4 — Supplementary Information 4. [file 41598_2023_45794_MOESM4_ESM.jpg]
